# Structure Refinement and Fragmentation of Precipitates under Severe Plastic Deformation: A Review

**DOI:** 10.3390/ma15020601

**Published:** 2022-01-14

**Authors:** Boris B. Straumal, Roman Kulagin, Leonid Klinger, Eugen Rabkin, Petr B. Straumal, Olga A. Kogtenkova, Brigitte Baretzky

**Affiliations:** 1 Karlsruhe Institute of Technology (KIT), Institute of Nanotechnology, Hermann-von-Helmholtz-Platz 1, 76344 Eggenstein-Leopoldshafen, Germany; roman.kulagin@kit.edu (R.K.); brigitte.baretzky@kit.edu (B.B.); 2 Chernogolovka Scientific Center of the Russian Academy of Sciences, Lesnaja Str. 9, 142432 Chernogolovka, Russia; kogtenkova@issp.ac.ru; 3 Department of Materials Science and Engineering, Technion—Israel Institute of Technology, Haifa 3200003, Israel; klinger@technion.ac.il (L.K.); erabkin@technion.ac.il (E.R.); 4 Baikov Institute of Metallurgy and Materials Science, Russian Academy of Sciences, Leninsky Prosp. 49, 119334 Moscow, Russia; straumal.peter@yandex.ru

**Keywords:** severe plastic deformation, dynamic equilibrium, steady-state, fragmentation nanocrystallization

## Abstract

During severe plastic deformation (SPD), the processes of lattice defect formation as well as their relaxation (annihilation) compete with each other. As a result, a dynamic equilibrium is established, and a steady state is reached after a certain strain value. Simultaneously, other kinetic processes act in opposite directions and also compete with each other during SPD, such as grain refinement/growth, mechanical strengthening/softening, formation/decomposition of solid solution, etc. These competing processes also lead to dynamic equilibrium and result in a steady state (saturation), albeit after different strains. Among these steady-state phenomena, particle fragmentation during the second phase of SPD has received little attention. Available data indicate that precipitate fragmentation slows down with increasing strain, though saturation is achieved at higher strains than in the case of hardness or grain size. Moreover, one can consider the SPD-driven nanocrystallization in the amorphous phase as a process that is opposite to the fragmentation of precipitates. The size of these crystalline nanoprecipitates also saturates after a certain strain. The fragmentation of precipitates during SPD is the topic of this review.

## 1. Introduction

All methods of severe plastic deformation (SPD), including ball milling (BM) [1], high pressure torsion (HPT) [2,3,4,5,6,7], planar twist channel angular extrusion (PTCAE) [8], equal channel angular pressing (ECAP) [4,9], equal channel angular pressing with the subsequent HPT (ECAP + HPT) [4], equal channel angular pressing with following cold rolling (ECAP + CR) [10], simple shear extrusion (SSE) [11,12], or constrained groove pressing (CGP) [13], have one common feature, namely in the SPD process, the deformed sample cannot be destroyed. In other words, the deformation increases, but the shape of the sample remains almost unchanged. This applies to both continuous SPD processes, such as BM or HPT, as well as to SPD processes that consist of repeating stages, such as ECAP, SSE, or CGP. It is clear that in the case of continuous processes, it is easier to observe the evolution of the structure and the properties of the sample. Obviously, with increasing deformation, the concentration of defects in the material increases. These are vacancies, interstitial atoms, dislocations, grain boundaries (GBs), and interphase boundaries (IBs). It is also obvious that the excess of accumulated defects cannot continue infinitely, although in some materials, it leads to the destruction of the crystal lattice, resulting in amorphization.

Simultaneously with the deformation and generation of excess defects, their relaxation processes also start, for example, the annihilation of vacancies and dislocations, the flux of interstitial atoms, or vacancies to dislocations and GBs, etc. With an increase in the number of excess defects, the rate of their relaxation and annihilation also increases until the rates of these two processes becomes equal. At this moment, a dynamic equilibrium between the formation and disappearance of defects is established, and the deformation continues in a steady state regime. In this steady state, many parameters of the material reach saturation. These include grain size [14,15,16,17,18,19,20,21,22,23], microhardness [8,15,23,24,25,26,27,28,29,30,31,32,33,34,35,36,37], and the lattice parameter in a solid solution [2,38,39,40,41]. A natural question arises: do all the parameters of the structure and properties of the material reach a steady state (saturation) simultaneously? This review is devoted to this issue. Here, we will pay special attention to the fragmentation of the second-phase particles as well as to the reverse process, namely the nucleation and growth of second-phase particles in the material under SPD.

## 2. Grain Size, Microhardness, Lattice Period

The simplest way to observe the dynamic equilibrium between defects production and their annihilation (relaxation) it is to measure the torsion torque during HPT. The torsion torque quickly increases after the beginning of HPT and then saturates after 1–1.5 anvil revolutions in Al-, Cu-, Mg-, Fe-, or Ti-based alloys as well as in high-entropy alloys [7,14,42,43,44,45,46,47,48,49,50,51,52,53,54,55,56]. For hard samples such as those of Nd-Fe-B alloys, the torsion torques saturates later, after 2–2.5 anvil rotations [42]. Other properties or structural parameters also saturate in the steady state. The most well-known example is the grain size decrease after SPD [14,17,18,19]. The grain size quickly decreases from several millimetres to a few hundreds of nanometers. Afterwards, the grain size reaches the steady-state and does not decrease anymore [15,20,21,22,23].

**Figure 1:**
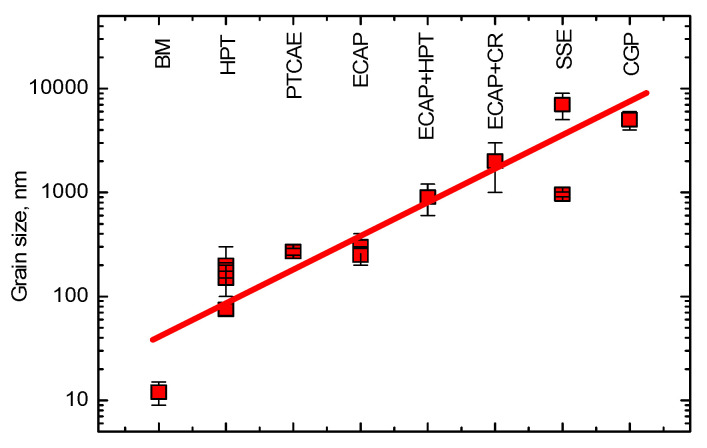
Steady-state grain size in copper subjected to the different SPD modes: 1—Ball milling (BM) [1], 2—HPT [2,3,4,5,6,7], 3—PTCAE [8], 4—ECAP [4,9], 5—ECAP + HPT [4], 6—ECAP + CR [10], 7—SSE [11,12], 8—CGP [13].

If we compare the steady-state grain size *d* for the same material (such as copper and copper-based alloys), we can see that it depends on the SPD mode. Thus, the minimum grain size *d* = ~15 nm can be achieved after ball milling (Figure 1) [1]. This is followed by HPT with *d* = 80–120 nm [2,3,4,5,6,7]. The *d* values for PTCAE [8], ECAP [4,9], ECAP + HPT [4,29], ECAP + CR [10], SSE [11,12], and CGP [13] are higher. The same tendency is also observed for aluminium and Al-based alloys [57].

The HPT temperature, pressure, and strain rate also influence the steady-state grain size *d* [42,43]. Thus, *d* decreases as the melting temperature increases (characterizing the strength of atomic bonds), decreasing the processing temperature and increasing the activation energy for self-diffusion and specific heat capacity [43,58]. The effective strain also plays an important role in reaching the dynamic equilibrium state. Thus, in case of HPT, the effective strain in the middle of the sample is always lower than it is in the sample periphery. As a result, the critical value of effective strain for the dynamic equilibrium is reached first at the periphery, where there is a lower effective strain that in the centre. For example, at a low number of anvil rotations, the grain size reaches its maximum in the middle, and the microhardness is at its minimum [15,25,26,27,28,29,30,31,32,33,34,35,36,37]. With increasing number of rotations, the difference between sample centre and periphery decreases. It totally disappears when the effective strain in the middle also reaches the critical value for the dynamic equilibrium.

**Figure 2:**
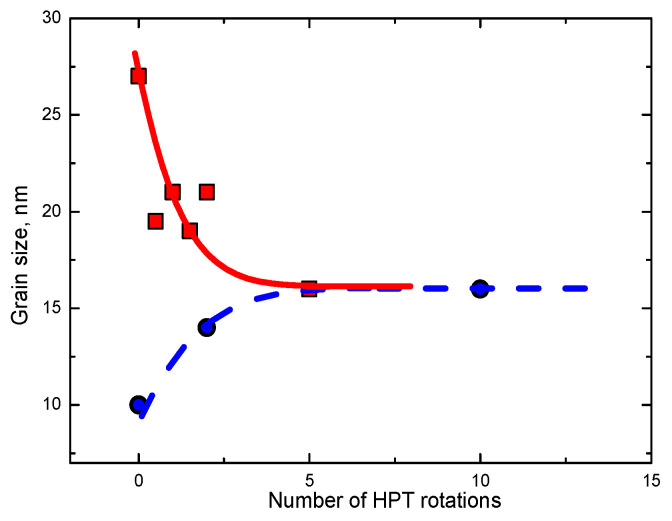
Grain size *d* after HPT plotted vs. number of rotations for pure coarse-grained steel sample (solid red squares) [60] and nanocrystalline steel produced by mechanical alloying (solid blue circles) [66].

It is important to say that the grain size is a real steady-state value in the dynamic equilibrium. This means that the steady-state *d* can be reached both “from the top” and “from the bottom”. For example, if one begins the HPT of the coarse-grained steel sample, one quickly reaches *d* = 15–20 nm [22,59,60,61,62,63,64,65,66] (Figure 2). On the other hand, if one starts the HPT of a steel sample produced by mechanical alloying with *d* = 10 nm, the grains do not become smaller. To the contrary, they grow up to the same steady-state value of 15–20 nm during SPD (Figure 2) [65]. A similar phenomenon of *d* decrease “from the top” and *d* increase “from the bottom” to the same steady-state value was observed also in nickel [67,68] and copper [69].

The explanation of this phenomenon is that the grain refinement processes compete with the nucleation and growth of new defect-free grains. If the grains are large in the initial state, then the intersection of dislocations, grain boundary sliding, deformation twining, etc., prevail at the beginning, and the grain size decreases. The driving force for the nucleation and growth of new defect-free grains is the decrease in the excess energy of the dislocations and interfaces disappearing during the growth of new grains. This driving force increases as the effective strain increases, and grain refinement slows down until the dynamic equilibrium is reached “from the top”. If the grains are large in the initial state, then they grow quick until the grain fragmentation mechanisms have enough space to work and to counteract the grain growth. Thus, the dynamic equilibrium is reached “from the bottom”.

**Figure 3:**
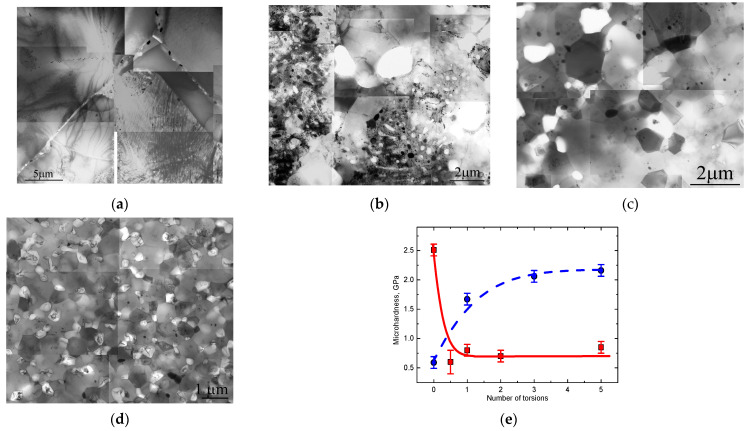
(**a**–**d**) Bright field transmission electron microscopy (TEM) micrographs of the Al–30% Zn alloy in the initial state (**a**) and after HPT: *n* = 0 (**b**), *n* = 0.5 (**c**) and *n* = 5 (**d**). (**e**) Vickers microhardness plotted vs. number of torsions for Al–30 wt.% Zn (solid red squares) [8] and Al–8.8 wt.% Mg alloys (solid blue circles) [25]. Reprinted with permission from ref. [8]. Copyright 2012 Elsevier. V.

SPD not only leads to quick grain refinement but also to changes in mechanical properties. For example, a large amount of experimental data has been collected on the Vickers microhardness during and after the SPD of Cu-, Al-, Ti, Mg-alloys, steels, etc., [15,23,30,31,32,33,34,35,36,37]. Normally, microhardness increases during SPD [15,23,30,31,32,33,34,35,36,37] and correlates with tensile strength [14,18,19,27,30,70,71,72]. The microhardness and tensile strength increase with the increasing rotation angle for HPT as well as with the increasing number of ECAP passes [32]. The mechanism explaining this phenomenon is the Hall–Petch hardening that is driven by grain refinement [73]. A few exceptions only underline this fact. Thus, the Hall–Petch hardening in Al–Zn alloys competes with solid solution softening due to the decomposition of the Al(Zn) solid solution. The grain size continuously decreases during HPT as usual (Figure 3a–d), but the alloy becomes softer (Figure 3e) [8,26].

The hardness and tensile strength also depend on the SPD mode and material, similar to the grain size. For example, if the purity of aluminium increases, then one can observe the transition from hardening to softening during HPT. Therefore, when the hardness of an alloy before SPD is higher than the steady-state value, then SPD would lead to its softening rather than its hardening [8]. Another example is the achievement of porosity saturation during the HPT of a powder material [74]. It is important to underline that different properties (torsion torque, grain size, resistivity [32], hardness, lattice parameter, etc.) do not reach the steady state simultaneously.

An interesting two-stage microhardness saturation can appear during composite deformation [75]. If the composite consists of two phases with different hardnesses, then the first steady state is reached when the microhardness of the softer phase saturates. If the shear stress (i.e., number of rotations in HPT) increases further, then the total microhardness increases again. It reaches the second steady state when the microhardness of harder phase saturates. The fact that the saturation in SPD is not always obvious can be due to similar inhomogeneities in the microstructure when different parts of the material reach the dynamic equilibrium at different effective strains.

Consider now the competition between dissolution and precipitation processes during HPT. In the steady state during HPT of a two-phase alloy containing the solid solution and precipitates of a second phase, a certain concentration in the solid solution *c*_ss_ is established. The *c*_ss_ value is controlled by the dynamic equilibrium between dissolution of a second phase and its precipitation. If the concentration in a solid solution *c*_init_ before HPT is below *c*_ss_, *c*_init_ < *c*_ss_, it would increase, and precipitates would dissolve during HPT. In another case, if *c*_init_ > *c*_ss_, then the concentration in a solid solution should decrease. As a result, the existing precipitates would grow (of new precipitates should form). This process is depicted as dynamic ageing [2].

The competition between the decomposition of the supersaturated solid solution and the dissolution of precipitates was investigated in Cu-based alloys [2]. Physically, a certain steady-state concentration *c*_ss_ in the solid solution is reached during SPD. Indeed, when one needs to compare different binary alloys with various maximal solubilities *c*_max_ in a solid solution, the model of a so-called effective temperature *T*_eff_ becomes quite useful. It means that after HPT, the concentration *c*_ss_ in a sample corresponds to an equilibrium solidus/solvus concentration at a certain elevated temperature *T*_eff_.

**Figure 4:**
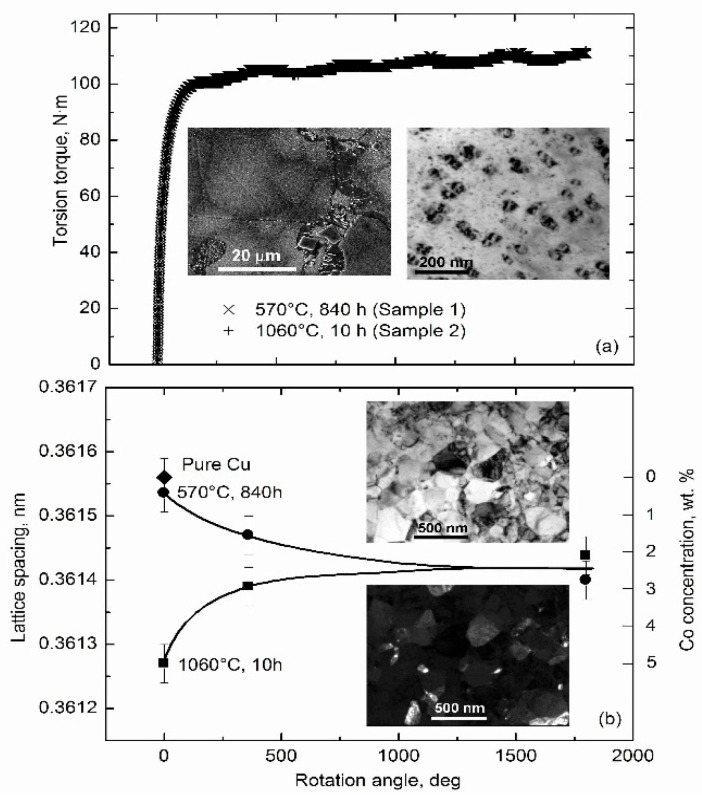
(**a**) Dependence of torsion torque on the HPT anvil rotation angle. The scanning electron microscopy (SEM, **left**) and transmission electron microscopy (TEM, **right**) micrographs in the insets are for the Cu–4.9 wt.% Co alloy after annealing at 570 °C for 840 h. (**b**) Dependence of lattice period on the HPT anvil rotation angle. Diamond shows the lattice period for pure copper. Circles mark the lattice period in sample annealed at 570 °C for 840 h. Squares correspond to the sample annealed at 1060 °C for 10 h. The respective cobalt concentration is shown on the right vertical axis, *c*_eq_ ≈ 2.5 wt.% Co. Insets: Bright-field (**top**) and dark-field (**bottom**) TEM micrographs of Cu–4.9 wt.% Co alloy after annealing at 570 °C for 840 h and HPT (6 GPa, 5 rot, 1 rpm). Reprinted with permission from ref. [2]. Copyright 2014 Elsevier.

As an example, we consider the Cu–Co system [2]. The grains in the Cu-based solid solution in the as cast Cu–4.9 wt.% Co alloy were of 10–20 μm in size, finely dispersed cobalt precipitates were of about 10–20 nm in size, and cobalt particles were of about 2 μm in size [2]. One sample was annealed at 1060 °C for 10 h. Co fully dissolved in the Cu-based matrix, and the (Cu) grain size grew in size to about 50 μm. Another sample was annealed at 570 °C for 840 h. Here, the Cu-based solid solution almost fully decomposed and less than 0.5 wt.%, and Co remained dissolved in Cu. After the HPT of both samples, the copper grain size decreased to ~200 nm, and the size of the Co-precipitates decreased to ~10–20 nm (see micrographs in Figure 4). The lattice parameter of the sample annealed at 570 °C before HPT was almost equal to that of pure copper (diamond in Figure 4). With an increasing number of anvil rotations, the lattice parameter of sample annealed at 570 °C decreased, and that of sample annealed at 1060 °C increased. After five anvil rotations, the lattice parameters in both samples became almost equal to each other and to that of the (Cu) solid solution with about 2.5 wt.% Co. The solid solution in both samples after HPT contained as much cobalt as if they would be annealed at *T*_eff_ = 900 ± 30 °C [2].

In [76], the values of *T*_eff_ are compared for several copper-based binaries, such as Cu–Co [2], Cu–Ni [77], Cu–In, Cu–Sn [38], Cu–Ag [39,40], Cu–Cr, Cu–Al–Ni, and Cu–Hf. The value of *T*_eff_ increases as the activation enthalpy increases for the bulk tracer diffusion *H*_D_ of a second component (Co, Ni, In, Sn, Ag, Cr, Al, or Hf). *T*_eff_ also linearly increases as the melting temperature *T*_m_ of Co, Ni, In, Sn, Ag, Cr, Al, or Hf increases as well. This is due to the correlation between *H*_D_ and *T*_m_ of the diffusing alloying component.

In ref. [78], the competition between the dissolution and formation of precipitates during SPD was investigated using molecular dynamics simulations. The case for interacting alloy components has been studied. Key findings of this work are the nucleation and growth of precipitates during SPD at a temperature of 100 K and Gibbs–Thomson-like behaviour relating steady-state solubility to precipitate size under sustained shearing. The direct relationship between effective temperature and the shear modulus was observed. The importance of cluster agglomeration during precipitate growth was underlined. The simulations provided good semi-quantitative agreement with experimental findings reported in the literature. In ref. [39], another model describing the dynamic equilibrium between dissolution and precipitation during HPT was proposed. Assuming that HPT fixes the composition at matrix–precipitate interfaces, it has been shown that the HPT-enhanced diffusive transport of species is the process that is likely controlling the observed steady-state composition in the matrix and the average diameter of the precipitates.

## 3. Fragmentation of the Second Phase Particles

If in the initial state, before the start of SPD, the sample is not single-phase, but consists of two or more phases, then with the onset of SPD, the fragmentation processes of particles in the second phase also starts. The most common variants of the initial structure of the material are (a) a mixture of powders of two phases, (b) a multilayer sample consisting of successive layers of two phases, and (c) compact samples in which the second phase is distributed in the form of particles in the volume or interlayers along the grain boundaries.

**Figure 5:**
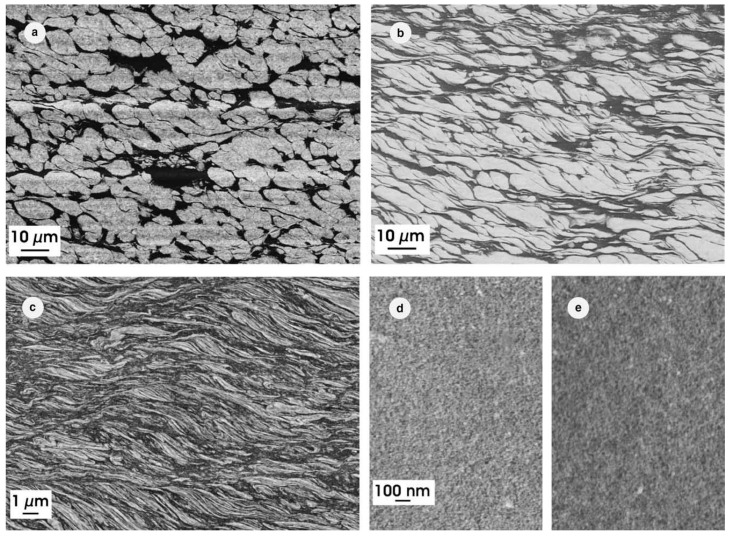
The microstructure of the W–25%Cu composite subjected to HPT at room temperature to strains ε of: (**a**) 4; (**b**) 16; (**c**) 64; (**d**) 256; (**e**) 512. Reprinted with permission from ref. [79]. Copyright 2005 Elsevier.

**Figure 6:**
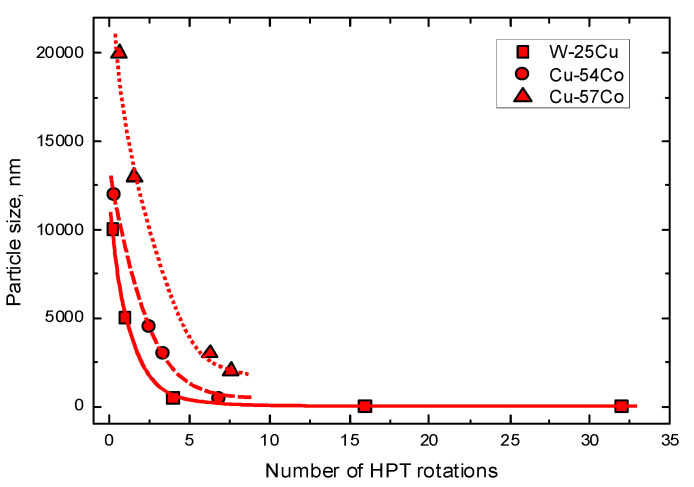
The dependence of particle size of on the number of HPT rotations. The size of W particles in the W—25 wt.% Cu alloy (solid red squares) was estimated from the data published in ref. [81]. The size of Co particles in the Cu—54 at.% Co alloy (solid red circles) and Cu—75 at.% Co alloy (solid red triangles) was estimated from the data published in refs. [82,83]. The lines are the guides for the eye.

In [79], the fragmentation process in the W–Cu alloy was studied. In the W–Cu system, the solubility of Cu in W as well as the solubility of W in Cu is negligible. The W—25 wt.% Cu nano-composite was fabricated from a mixture of powders of two phases Cu and W with a grain size of 2–10 μm using HPT. The samples were deformed at a pressure of 8 GPa, and they had a diameter *R* of 8 mm and a thickness *t* of 0.8 mm. The number of turns *n* was selected to attain a certain equivalent strain at a radius *r* according to the relation ε = 1.15 π*nrt*^−1^. As a result, after 10 turns, the homogeneous microstructure with a W particle size of 10–20 nm was obtained [80]. Figure 5 demonstrates how the size of the W grains decreases and reaches the steady state value. We estimated the grain size of W particles from the data published in ref. [81] and plotted it in Figure 6 against the number of HPT rotations *n* (solid red squares). In [81], the same W—25 wt.% Cu powders were deformed not only at room temperature but also at 200 °C and 400 °C. The increase in the HPT temperature slows down the fragmentation process. This is because the rate of relaxation processes increases with increasing temperature, while the plastic deformation work achieved by the HPT tool remains approximately constant.

In [82,83,84], the fragmentation process was studied for the Co–Cu alloys with 25, 54, and 75 at.% Co for Co–Cu with small mutual solubility. At peritectic temperature, about 9 at.% Co is solvable in Cu, and about 12 at.% Cu is solvable in Co. Similar to refs. [79,81], the starting state was the mixture of powders of the two phases of Cu and Co, with a grain size of 2–10 μm. The microhardness values saturated at ε = ~100 (about 4.5 turns). In Figure 6, the dependence of the Co grain size on number of turns is shown (solid red circles for Cu—54 at.% Co and solid red triangles for Cu—75 at.% Co). The fragmentation of the Co phase proceeds more slowly in the Cu—75 at.% Co alloy. This is easy to understand due to the higher strength of Co in comparison with Cu. Recently, we observed the dissolution of about 2.5 at% Co in the copper matrix [2] during HPT. Through the HPT of Cu–Ag alloys, not only silver dissolves in the copper matrix, but Cu also dissolves in Ag particles [40,85]. Therefore, one can suppose that during the HPT of mixture of Cu and Co powders in [82,83,84], the fragmentation also took place with the mutual dissolution of copper and cobalt. This could be the reason why the fragmentation process of Co–Cu alloys goes slower in comparison to W–Cu ones (see Figure 6).

**Figure 7:**
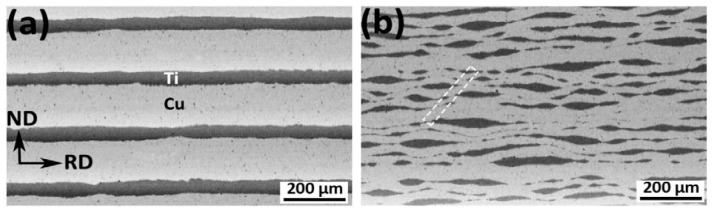
SEM micrographs of the ARB processed Cu/Ti bimetal composites after (**a**) 1 cycle, (**b**) after 3 cycles. Reprinted with permission from ref. [86]. Copyright 2017 Elsevier.

**Figure 8:**
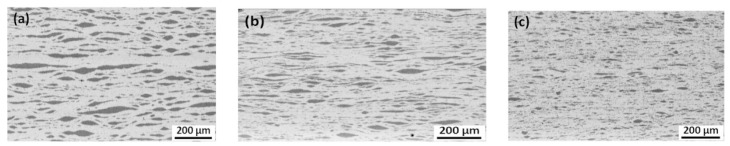
SEM micrographs of the ARB processed Cu/Ti bimetal composite after (**a**) 5 cycles, (**b**) 7 cycles, and (**c**) 9 cycles. Reprinted with permission from ref. [86]. Copyright 2017 Elsevier.

In ref. [86], a Cu/Ti bimetal nanocomposite was manufactured by accumulative roll-bonding (ARB). In their original state, the commercially pure copper and titanium annealed sheets with an initial thickness of 300 and 100 μm, respectively, were scratch-brushed by a circular stainless steel brush. Afterwards, six Cu and five Ti strips were stacked alternatively together in an initial sandwich, with 22 vol.% Ti having an overall thickness of 2.3 mm. After that, it was roll-bonded to prepare a multilayered composite with a thickness of 1 mm. This specimen was denominated as a first cycle ARB processed sample (Figure 7a). It is essential that the thickness reduction in this roll-bonding step is over 50%. It guarantees the formation of acceptable bonding between the copper and titanium layers. The ARB process was then repeated up to nine cycles, with a ~50% thickness reduction after each pass. During further ARB, the Ti layers break and form the elongated particles (see Figure 7 and Figure 8).

**Figure 9:**
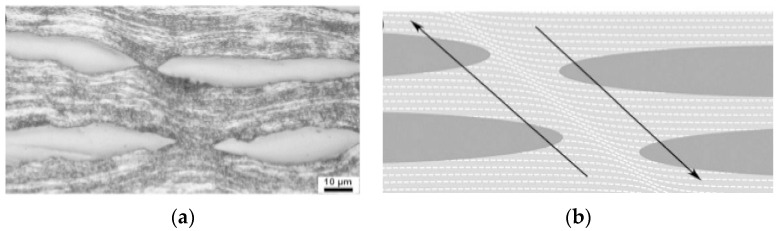
(**a**) Shear band formation within the composite after three ARB cycles. (**b**) Schematic illustration of segmentation mechanism at a shear band. Reprinted with permission from ref. [86]. Copyright 2017 Elsevier.

The model for the mechanical fragmentation of Ti layers has been proposed [86]. The fragmentation mechanism was described as follows: In the first ARB cycle, the presence of active crystallographic slip systems and the low density of dislocations in the annealed Cu and Ti structure result in the proper ductility and uniform deformation of both layers. However, with further processing, the dislocation density increases. An increase in the mechanical strength follows as well as a sharp decrease in ductility in comparison with materials after annealing. Such work-hardening by hindering in dislocation motion leads to the formation of shear bands (see Figure 9a). Such shear bands are the localized deformation zones. Consequently, the necking and fragmentation of Ti layers happen at these locations (see Figure 9b), where the geometrical softening overcomes the work hardening rate. In case of HPT, the fragmentation mechanism of the initial layered structure differs from that for ARB. In particular, the typical “torques” appear at a certain intermixing stage [16]. 

**Figure 10:**
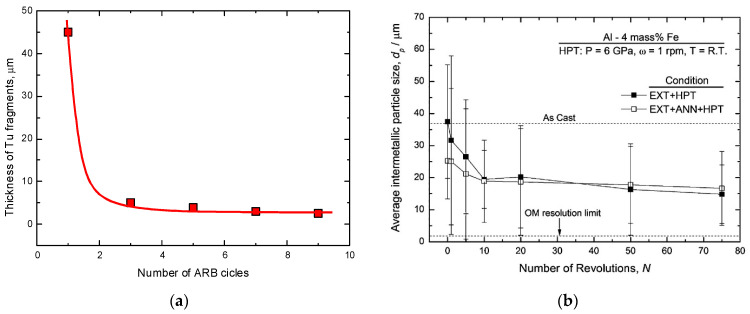
(**a**) Dependence of mean thickness of Ti layers/fragments within the Cu matrix on the number of ARB cycles estimated from the data published in refs. [87,88,89,90,91,92,93]. The lines are the guides for the eye. (**b**) The dependence of size *d* of Al_3_Fe intermetallic particles in the Al—4 wt.% Fe alloy on the number of HPT rotations *n*. *d*(*n*) curves are shown for two cases, namely for the samples after extrusion and annealing followed by HPT (EXT + ANN + HPT, open squares) and for the alloys after extrusion followed by HPT (EXT + HPT, solid squares). Reprinted with permission from ref. [90]. Copyright 2012 Elsevier.

**Figure 11:**
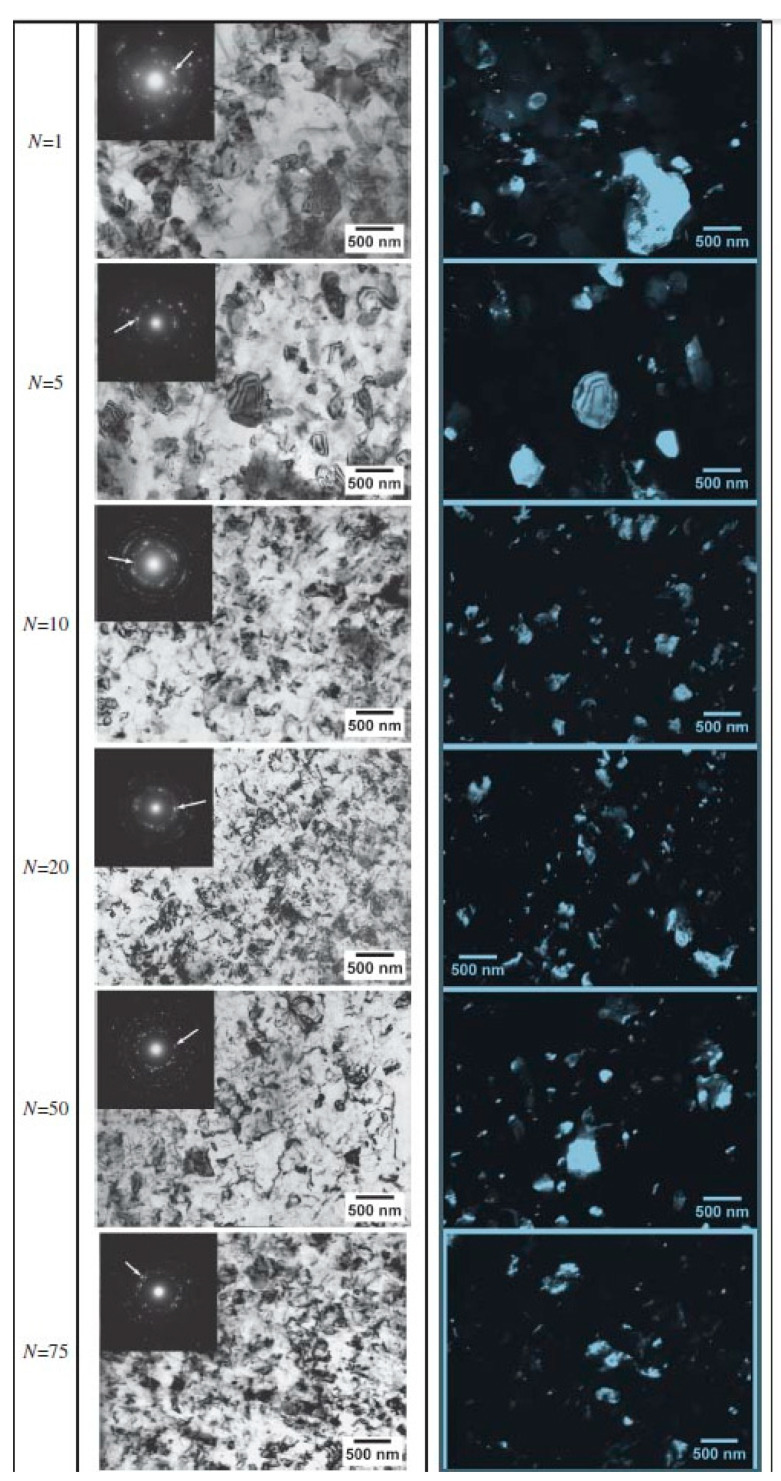
TEM micrographs showing Al—4% Fe EXT + HPT microstructure evolution for different number of anvil rotations *n*. Dark field images obtained from diffracted beams indicated by arrows in corresponding SAED patterns (as insets in bright field images). Reprinted with permission from ref. [90]. Copyright 2012 Elsevier.

Figure 10a shows the dependence of mean thickness of Ti layers/fragments within the Cu matrix on the number of ARB cycles estimated from the data published in refs. [87,88,89,90,91,92,93] (see Figure 11). We see that the thickness of the Ti fragments obviously saturates and arrives at the steady-state value of about 2 μm. This value is much higher than the steady-state fragments size in W–Cu and Co–Cu alloys after HPT (see Figure 6). This is most likely due to the difference in mechanical conditions for the fragmentation between ARB and HPT processes.

One can find important examples of second-phase fragmentation in the papers devoted to the SPD-driven fragmentation of cementite inside carbon steels [60,61,62,63,64,65,87,88]. The cementite plates are first broken, spheroidized, and in some cases, almost disappear. The newly formed grain boundaries (GBs) play an important role in this process. The amount of cementite decreases in the steel because the carbon forms the segregation layers in the new GBs during SPD-forced grain refinement [89]. These layers “consume” the carbon from cementite particles and decrease their volume fraction.

In ref. [90], a bulk Al—4 wt.% Fe alloy processed by extrusion or by a combination of extrusion (EXT) and successive annealing was subjected to HPT at 6 GPa, 1 rpm, and for up to 75 plunger revolutions. After extrusion and successive annealing the samples contained the (Al) matrix (appears black in the dark-field TEM images, Figure 11) with Al_3_Fe intermetallic particles (appears bright in the dark-field TEM images, Figure 11). These Al_3_Fe particles continuously fragmented during HPT. The Al_3_Fe size saturates at ~20 μm after about 10 plunger revolutions and remains unchanged afterwards (Figure 10b). The Vickers microhardness also saturates but later, after about 20 plunger revolutions. The grain size in (Al) matrix is about 250 nm, which is typical for the Al-based alloys after HPT. It reaches the saturation between one and five revolutions. Thus, the steady-state size of Al_3_Fe particles during HPT [90] is at least two orders of magnitude higher than that in Cu alloys with W, Co, and Ti after HPT and ARB [81,82,83,86]. This striking difference in fragmentation behavior can be attributed to a number of factors, such as different volume fractions of the particle phase, different hardness ratios between the particles and the matrix, and differences in the energies between the GBs and IBs. For example, in the W-Cu system, the Cu may wet the GBs in W, thus preventing the agglomeration of fragmented W particles.

The important point is, whether the composition or phases are changing in the particles during their fragmentation. The composition changes during SPD cannot be observed in the stoichiometric compounds (or daltonides) which exist in the very narrow composition interval [60,61,62,63,64,65,87,88,89,90]. In this case, only the portion of such compounds can be measured, and its possible change could be observed, such in the case of cementite in steels [89]. If the intermetallics exist in broad composition intervals (so-called bertollides), such as in the Hume–Rothery phases, such composition changes can be measured. However, some indications of the peak shift for the ε-phase in Cu–Sn alloys can be found in the XRD diffraction patterns measured with synchrotron light before and after HPT [38]. The HPT-driven change of the composition was also observed in the silver particles distributed in the Cu-matrix [40]. Before HPT, the Cu-matrix was almost free from silver, and the silver particles also contained almost no copper. However, after HPT, a steady state was reached that was equivalent to the effective temperature *T*_eff_ = 600 °C. In other words, the silver concentration in the copper matrix as well as the copper content in the silver particles increased and reached values equal to the solubilities at *T*_eff_ = 600 °C.

## 4. Nanocrystallization of Amorphous Alloys and the Growth of Particles of the Second Phase

In Section 2, we that in most cases, SPD leads to grain refinement in the material until the grain size drops to a certain steady-state value. However, in a number of experiments, it was possible to fabricate the original samples (before SPD) with a grain size smaller than the steady-state one. Then, the grain size, on the contrary, increased with the SPD until it reached the same stationary value (see Figure 2).

Unfortunately, at present, to the best of our knowledge, there are no observations of processes inverse to the fragmentation of second-phase particles. On the other hand, there are a number of studies in which there are no crystals at all in the initial state before SPD. In these studies, samples are subjected to SPD deformation in an amorphous state. This amorphous state is obtained using conventional amorphization technologies, such as rapid solidification. The data for the nanocrystallization during HPT of amorphous ribbons (AR), bulk amorphous alloys (BA), and helium gas atomized samples (He) are given in Table 1.

**Figure 12:**
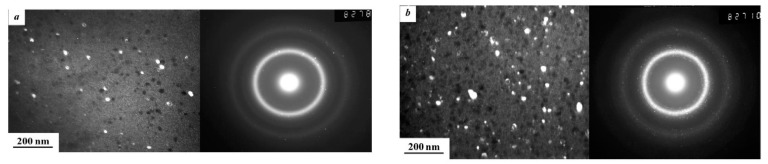
Dark-field images of the Fe_53.3_Ni_26.5_B_20.2_ amorphous ribbons structure subjected to HPT to *n* = 3 (**a**) and *n* = 9 (**b**) and the corresponding selected area electron diffraction (SAED) patterns. Reprinted with permission from ref. [106]. Copyright 2020 MDPI.

**Figure 13:**
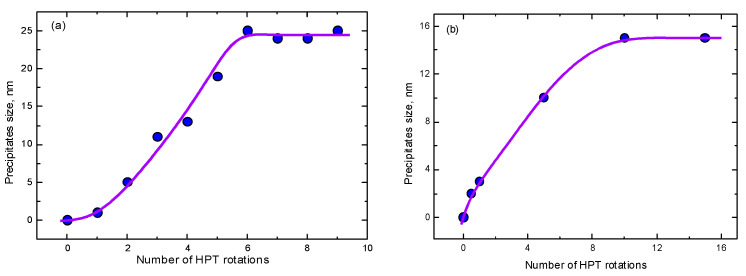
The dependence of size of nanocrystals (**a**) in the Fe_53.3_Ni_26.5_B_20.2_ amorphous alloy estimated from the data published in ref. [106] and (**b**) in the Ti_50_Ni_25_Cu_25_ amorphous alloy estimated from the data published in refs. [107,108] on the number of HPT rotations. The lines are the guides for the eye.

Thus, in the majority of cases, the nanocrystallization was only investigated for one strain value (most frequently equivalent to *n* = 5). However, there are at least three examples where the nanocrystallization was studied for several strain values. In the first case, the Fe_53.3_Ni_26.5_B_20.2_ and Co_28.2_Fe_38.9_Cr_15.4_Si_0.3_B_17.2_ (at. %) 25 μm thick amorphous ribbons quenched from the melt were studied [106]. The compacted disks made of these ribbons were subjected to HPT at 6 GPa with a rotation rate of 1 rpm for 0.5 to 9 anvil rotations. At the beginning, the samples were completely amorphous, i.e., the size of the crystals was zero. Then, during HPT, the nanograins belonging to the crystalline phase started to nucleate in amorphous matrix (Figure 12). In Figure 13a, the size of the nanocrystals in the Co_28.2_Fe_38.9_Cr_15.4_Si_0.3_B_17.2_ alloy estimated from the data published in ref. [106] is plotted against rotation number *n*. First, the nanocrystals appeared already after 1 rotation, then the size of the nanocrystals increased and saturated at ~25 nm after about six anvil rotations. In the Fe_53.3_Ni_26.5_B_20.2_ and alloy of the first nanocrystals appeared after three rotations, and their size saturated at about 40 nm (see Figure 11). When the size of the nanocrystals saturated, their amount continued to increase (compare number of bright spots in SAED patterns in Figure 12a,b) [106].

In Refs. [107,108], the Ti_50_Ni_25_Cu_25_ amorphous alloy obtained by the melt spinning were studied. Its HPT took place at 7 GPa, 1 rpm with *n* = 0.5, 1, 5, 10, and 15 anvil rotations. The first nanocrystals had already appeared after *n* = 0.5. They are not equiaxial as in [106] but elongated. In Figure 13b, the thickness of the nanocrystals in the Ti_50_Ni_25_Cu_25_ alloy estimated from the data published in refs. [107,108] is plotted against rotations number *n*. The size of these nanograins of B2 phase crystallized from the amorphous matrix under the action of HPT saturated at about 15 nm.

**Figure 14:**
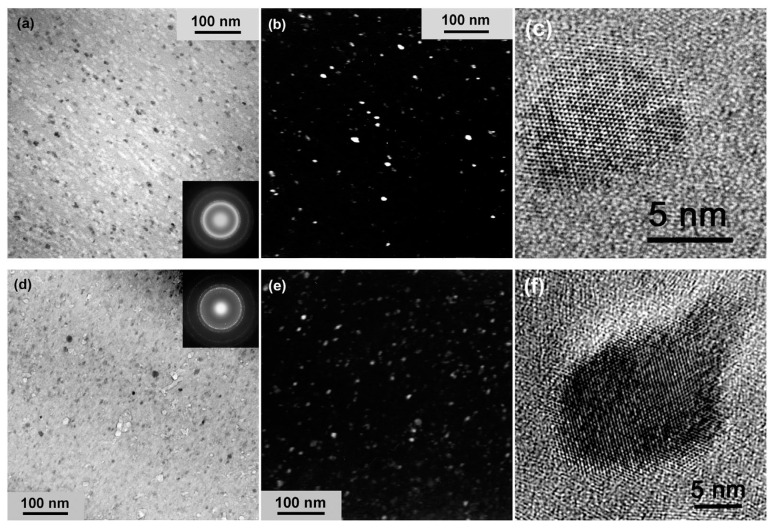
Microstructure of the Al_90_Y_10_ alloy subjected to deformation at *n* = 0.1 (**a**–**c**) and *n* = 2 (**d**–**f**). (**a**,**d**)—BF images, (**b**,**e**)—DF images, inserts—corresponding SAED patterns, (**c**,**f**)—HRTEM images of individual Al crystals having formed after deformation. Reprinted with permission from ref. [94]. Copyright 2017 Elsevier.

**Figure 15:**
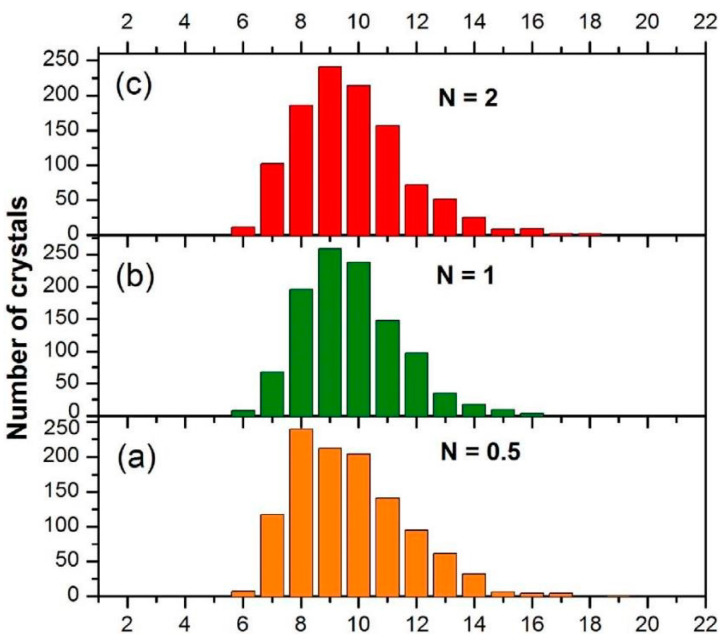
Size distribution for Al_90_Y_10_ alloy subjected to HPT at the strain corresponding to (**a**) *n* = 0.5, (**b**) *n* = 1, and (**c**) *n* = 2. Reprinted with permission from ref. [94]. Copyright 2017 Elsevier.

In Refs. [93,94], the HPT of amorphous ribbons of Al_90_Y_10_ alloy was studied for *p* = 5 GPa, 1 rpm, *n* = 0.1, 0.5, 1, 2 and 5. The extremely fine Al particles crystallized in the amorphous matrix (see TEM micrographs in Figure 14). First, the Al nanocrystals are visible even after 0.1 plunger revolutions (Figure 14a). Moreover, their size had already saturated at ~9 nm after 1 plunger revolution (Figure 15).

The formation of nanocrystals from an amorphous matrix during HPT has another important aspect in addition to the size of these nanocrystals. When studying the competition between decomposition and formation of solid solutions during HPT, we found that the composition of the solid solution converges to a certain stationary value of *c*_ss_ [2,41]. This steady-state value is the same as in solid solutions equilibrated at a certain elevated temperature. This temperature is commonly called the effective temperature *T*_eff_. Apparently, a certain physical meaning is hidden behind the formal similarity of the concentration in the solid solution at HPT and the concentration in the solid solution after annealing at the effective temperature *T*_eff_. In other words, under severe external action, a certain increased steady-state concentration of lattice defects is formed. This increased concentration of lattice defects is similar, for example, to a high vacancy concentration at an elevated temperature *T*_eff_. Moreover, we found that the value of the effective temperature in copper alloys is proportional to the height of the energy barrier for bulk diffusion in these alloys. This means that the higher the energy barrier for diffusional mass transfer, the slower the relaxation of defects arising during deformation, and the higher the steady-state concentration of excess defects, and hence the effective temperature *T*_eff_. The value of *T*_eff_ can be determined not only from the value of the concentration in the solid solution, but also from the temperature range of the existence of certain intermetallic phases. Such a possibility also exists during nanocrystallization analysis.

For example, in refs. [98,109,110], the amorphous alloy Al_85_Ce_8_Ni_5_Co_2_ was subjected to three types of processing: equilibrium annealing, high pressure torsion, and ball milling. Ball milling leads to the formation of a mixture of α-Al and Al_11_Ce_3_ phases. This is equivalent to the annealing at *T*_eff_ = ~560 K. During HPT, only the α-Al phase was formed in the alloys. This is equivalent to the annealing in the temperature range 535–543 K. Thus, the effective temperature for HPT, which can be determined from the data on nanocrystallization in the Al_85_Ce_8_Ni_5_Co_2_ alloy, is approximately *T*_eff_ = 540 K. It is below the *T*_eff_ for ball milling. This fact correlates with the data shown in Figure 1, which indicates that the steady-state grain size for ball milling is lower that the steady-state grain size for HPT. In other words, the steady-state concentration of excess defects is higher for ball milling compared to HPT.

## 5. Conclusions

1. The various SPD methods lead to the steady state microstructure and properties after certain strain value. This is because of the dynamic equilibrium between the deformation and relaxation processes.
2. The steady state of different properties is achieved at different strain values and can be reached both “from the top” and “from the bottom”. We briefly discussed the examples of well-known steady state for torsion torque at HPT, grain size, microhardness, and concentration in solid solution.
3. The main topic of this review was, however, the possible competition between the fragmentation of second phase particles and their growth. The direct experimental observations of competition between fragmentation and growth are still absent. Therefore, we compared the decrease in the second phase particles during HPT and ARB (as “from the top” process) with the growth on nanocrystals in the amorphous matrix (as “from the bottom” process).
4. It looks like the saturation value of particle size depends on their hardness and volume fraction. The hard and brittle particles remain (or become) bigger than the soft and deformable ones. Certainly, the main fragmentation mechanism is the mechanical rupture by the shear bands crossing the particles. In the presence of mutual solubility, the mechanism of competing dissolution/precipitation also influences the fragmentation/growth process.

## Figures and Tables

**Table 1:** Nanocrystallization during HPT of amorphous ribbons (AR), bulk amorphous alloys (BA), and helium gas atomized sampl (He).

Alloy	Grain Size of Nanocrystals, nm	HPT Pressure *p*, GPa	Anvil Rotation Rate, rpm	Anvil Rotation Number, *n*	Reference
Al_88_Y_7_Fe_5_	12	6	1	5	[91] AR
Al_85_Y_8_Ni_5_Co_2_	fcc Al, 13	6	1	5	[92] AR
Al_90_Y_10_	7	5	1	5	[93] AR
Al_90_Y_10_	10	5	1	0.1, 2	[94] AR
Al_85_Ce_8_Ni_5_Co_2_	19	6	1	5	[95,96] AR
Fe_78_Si_13_B_9_	6	4	1	5	[97] AR
Nd_9_Fe_85_B_6_	<10	6	1	5	[98,99] AR
Cu_60_Zr_20_Ti_20_	<20	6	1	5	[100] AR
Ti_50_Ni_25_Cu_25_	~20, *T* = 20 °C	6	1	10	[101] AR
Ti_50_Ni_25_Cu_25_	~20, *T* = 150 °C	6	1	10	[102] AR
Ti_50_Ni_20_Cu_30_	~20–100	6	1	1, 3, 5	
Vitreloy Zr_44_Ti_11_Cu_10_Ni_10_Be_25_	10, *T* = 610, 620, 630 K	8	0.2	1	[103] BA
Zr_65_Cu_17_Ni_5_Al_10_Au_3_	50	5	2	1	[104] BA
Al_85_Ni_10_La_5_	10	6	0.3	1	[105] He

## Data Availability

Data are contained within the article.
